# Commentary: Maintaining well-being during COVID-19: a follow-up study of community dwelling older people in New Zealand

**DOI:** 10.1177/17449871251347333

**Published:** 2025-07-02

**Authors:** Julie Cooke

**Affiliations:** Senior Lecturer Adult Nursing Faculty of Health, Science and Technology Oxford Brookes University, Oxford, UK

Waterworth and Raphael (2025) present an interesting contribution to managing well-being during a global pandemic from the perspective of older people in New Zealand. As a nurse academic and early career researcher, several profound points came to mind whilst reading this work. This commentary will now focus on three of those key aspects, which will be of interest to policymakers, nurse educators and clinicians: the concept of healthy ageing in society, whether or not healthcare definitions of ‘old’ remain adequate for today’s population and their healthcare needs, and the impact of ageism on older adults’ ability to feel valued by society.

The World Health Organization (WHO, 2024) reports that global life expectancy has seen a slow but steady increase since the 1950s. According to the Office for National Statistics (ONS, 2024), the average life expectancy for a person born in 2022 is 90 years, compared to 62 years for men and 74 years for women born in 1951 in the United Kingdom (UK). This is reflected worldwide, with the United Nations (UN, 2020) predicting the largest projected increases in life expectancy in developing countries in Northern Africa and Asia. The WHO (2024) predicts that by 2050, three times as many people aged over 80 will be living with, managing, and dying with complex multiple comorbidities. Although this study interviewed people aged 75+, the age of 65+ is commonly accepted in the healthcare literature and creates some debate about the appropriateness of the current definition of older age. For example, in the UK, the ONS (2024) defines old age as 65+, whereas the WHO (2024) and the UN (2023) describe an older person as over the age of 60. Additionally, a new definition to identify people over the age of 85 has become known as the ‘oldest old’ as described by Age UK (2012), and suggests that some of these people will not be dependent at all. These current definitions, that older people are those over 60 years of age into account (ONS, 2024), mean that ‘old’ currently spans over a quarter of the average life expectancy.

A seminal finding of the study was that older people valued being provided with accurate information and concluded that this is critical for health promotion communication. Kagan (2009) suggests that the current definitions of ‘old age’ have led to the creation of boundaries that result in more generalist than specialist knowledge, resulting in a poor understanding of the needs of the older population by healthcare professionals. This lack of understanding within healthcare for the needs of older people, Shneerson et al. (2014) suggested, could lead to limited funding for resources and poor awareness and communication of the available health promotion services for people to age well in society. In 2022, Kagan further argued that the continued repetitive presentation of acutely unwell older people aged over 65 within healthcare settings could lead us to believe that all older people have these needs, which promotes implicit ageism. My doctoral thesis exploring nurse educators’ experiences and perceptions of supporting nursing home placements concurred with Kagan’s (2022) argument and added that implicit ageism, which could be promoted by age definition, also impacts the provision of nursing older people within nurse education (Cooke, 2024).

Healthcare providers should be aware of the impact of ageism, implicit or otherwise, and its detrimental effect on social isolation and older people’s emotional well-being and ability to feel valued in society. One of the key findings of (Waterworth and Raphael, 2025) was that expressing gratitude for not only receiving support, but also the ability to provide support to others and ‘do their bit’ for their country, mattered to the participants and improved their sense of value and well-being. Older people have a wealth of experience and contribute in many ways to their families’ and communities’ everyday lives. Despite this, older people are often assumed to be a ‘burden’ on society (WHO, 2024). The UN Decade of Healthy Ageing (2021–2030), led by the WHO, strives to address the health inequalities faced by older people by challenging society to change how they think, feel, and act towards age and ageism (WHO, 2024). Additionally, Nathoo (2017) argued that compassion is an important element of healthcare that promotes a sense of well-being and belonging. This suggests that if both the government and wider society accept responsibility for addressing the stigmatisation afforded to older people through the introduction of new policy reform, and invite the contributions of older people, this will increase the resources and support available to enable ageing well in society.

In summary, this study offers the unique insights of older people to explore how they managed their well-being during the COVID-19 pandemic in New Zealand and offers important points for consideration by policy, practice and research. Many interesting and important topics for discussion have arisen as a result, which can now be debated by governments, professionals, charities, academics and the media to develop collaborative global action to foster longer and healthier lives.
